# Impact of a 24-Week Mobile App–Based Human Coaching Program on Body Composition and Lipid Metabolism in Breast Cancer Survivors With Overweight or Obesity: Single-Arm Prospective Cohort Study

**DOI:** 10.2196/64846

**Published:** 2025-09-24

**Authors:** Eun-Gyeong Lee, Dong-Eun Lee, Jeongseon Kim, Jaihong Han, Seeyoun Lee, Han-Sung Kang, Eun Sook Lee, Heejung Chae, Sung Hoon Sim, Keun Seok Lee, Jungeun Lee, Hyun Jeong Lee, Ji Sung Yoo, Gyung Ah Wie, So-Youn Jung

**Affiliations:** 1 Department of Cancer Biomedical Science, Graduate School of Cancer Science and Policy, National Cancer Center, Goyang, Republic of Korea; 2 Center for Breast Cancer, National Cancer Center, 323 Ilsan-ro, Ilsandong-gu, Goyang, 10408, Republic of Korea, 82 31-920-1681, 82 31-920-2799; 3 Biostatistics Collaboration Team, Research Core Center, Research Institute of National Cancer Center, Goyang, Republic of Korea; 4 Noom, Inc., New York, NY, United States; 5 Department of Psychiatry and Behavioral Science, National Cancer Center, Goyang, Republic of Korea; 6 Department of Rehabilitation Medicine, Research Institute and Hospital, National Cancer Center, Goyang, Republic of Korea; 7 Department of Clinical Nutrition, National Cancer Center, Goyang, Republic of Korea

**Keywords:** mobile app, body weight, survivor, breast cancer, obesity

## Abstract

**Background:**

Overweight or obesity is a prognostic factor for breast cancer recurrence and breast cancer–related deaths. However, weight control is difficult for breast cancer survivors because of menopause, chemotherapy, antihormonal therapy, and psychological issues.

**Objective:**

This study aimed to develop a 24-week mobile app–based human coaching program using Noom and evaluate its efficacy in breast cancer survivors who are excessively overweight or with obesity, including those who successfully used the program.

**Methods:**

In this single-arm prospective cohort study, 130 breast cancer survivors with BMI ≥25 were enrolled and received a 24-week program, including diet-, exercise-, and psychology-based content with the trained human coach in Noom between 2019 and 2021. For a hyperactive group who joined for more than 16 weeks, we evaluated weight, BMI, lipid level, bioimpedance, and quality of life at baseline, 6-month, and 12-month follow-up.

**Results:**

Among 130 breast cancer survivors, 101 (77.7%) and 93 (71.5%) completed the 6-month and 12-month follow-ups, respectively. The mean age of all participants was 54.90 (SD 7.42) years. At baseline, the median BMI was 27.14 (IQR 25.20‐35.36) for the hyperactive group and 27.50 (IQR 25.20‐35.50) for the active and inactive group. In the hyperactive group (68/101, 67%), body weight and BMI significantly reduced (mean difference −1.97, 95% CI −2.65 to −1.26 kg; *P*<.01 and mean difference −0.86, 95% CI −1.15 to −0.56; *P*<.01, respectively) at 6 months and were maintained at 12 months without the yo-yo effect. Among the lipid panel, triglyceride levels decreased significantly (−34.13, 95% CI −58.09 to−10.17; *P*<.01) and were maintained at 12 months. With respect to bioimpedance components, skeletal muscle mass (kg), body fat mass (kg), percent body fat (%), waist-to-hip ratio, and visceral fat area (cm^2^) improved in the first 6 months. However, waist-to-hip ratio and visceral fat area increased during the next 6 months. Based on the European Organisation for Research and Treatment of Cancer Quality of Life Questionnaire-Core 30 (EORTC QLQ C30) and Breast Cancer Module (23 items), nausea or vomiting, constipation, body image, and arm and breast symptoms significantly improved during the first 6 months.

**Conclusions:**

This study demonstrated that a 24-week mobile app–based human coaching program is beneficial for controlling body weight, BMI, triglyceride, and body composition in terms of bioimpedance for breast cancer survivors who are excessively overweight or have obesity.

## Introduction

Obesity is associated with the risk of breast cancer and its recurrence. This relationship involves altered fatty acid metabolism, extracellular matrix remodeling, secretion of adipokines and anabolic and sex hormones, immune dysregulation, and chronic inflammation [1]. A previous systematic meta-analysis showed that women with obesity and breast cancer had poorer survival rates than women without obesity [2].

This increase in the number of breast cancer survivors is largely attributable to advancements in early detection and treatment. An estimated 297,790 new cases of invasive breast cancer are expected in women in the United States, along with 55,720 new cases of noninvasive breast cancer. There are over 3.8 million breast cancer survivors in the United States. The 5-year relative survival rate for patients with breast cancer is 91% [3]. Breast cancer survivors face significant challenges in managing their weight because of a confluence of factors, including hormonal changes induced by menopause, side effects of chemotherapy and antihormonal treatments, and psychological stressors related to their condition. It is crucial to reduce BMI and maintain an optimal BMI in women with obesity and breast cancer.

Exercise interventions have gained increasing attention globally for their role in improving body composition, physical fitness, and overall health outcomes among individuals with overweight and obesity, including breast cancer survivors [4]. Among various modalities, combined aerobic and resistance training is highly effective in enhancing body composition, lipid metabolism, inflammation, and overall quality of life in this population [5]. In addition, high-intensity interval training and combined aerobic and resistance training have been shown to provide both physiological and psychological benefits, including improvements in cardiometabolic markers, mood, and exercise adherence. Furthermore, these structured exercise programs are safe and effective in populations with metabolic diseases, reinforcing their role in weight management strategies [6,7].

Noom is a digital app dedicated to weight management that features functionalities for monitoring caloric intake and physical activity, facilitating collective encouragement through group involvement, and crucially providing individualized coaching. Unlike programs that prioritize rapid weight reduction, this platform educates users on acquiring new competencies and establishing sustainable healthy practices. Such mobile apps are increasingly used in the health care sector. Comparing the dietary data collected on energy and macronutrients, Noom’s estimates for mean daily fat intake and percent total energy from carbohydrates are comparable to those of conventional dietary assessment tools. Noom’s estimates for daily energy, protein, and carbohydrate intakes are significantly higher. The Noom could be useful for monitoring dietary intake, although more research is needed to determine its accuracy for micronutrients and other dietary components [8]. A study of 35,921 Noom Coach app users found that 77.9% experienced weight loss, with dinner input frequency being the most significant factor (odds ratio [OR] 10.69, 95% CI 6.20-19.53; *P*<.001), and frequent weight input reducing the yo-yo effect (OR 0.59, 95% CI 0.39-0.89; *P*<.001). This study demonstrates the app’s clinical utility for weight reduction, especially for users who consistently monitor their weight and diet [9].

This study aimed to develop a 24-week mobile app–based human coaching program using Noom and evaluate its efficacy in breast cancer survivors who are excessively overweight or with obesity.

## Methods

### Trial Design and Study Population

We conducted a single-arm prospective cohort study between 2019 and 2021 at the National Cancer Center in the Republic of Korea. We provided an informational paper tailored to breast cancer survivors at the National Cancer Center within the existing Noom program. Experts in breast cancer, rehabilitation, nutrition, and psychiatry contributed to the writing of this paper. We planned to enroll breast cancer survivors who were at an excessive weight or with obesity. They were enrolled and joined a 24-week mobile app–based human coaching program during the first 6 months. They were followed up for 12 months without further application of the Noom program. Participants were recruited via physician referrals during routine visits to the outpatient breast cancer center.

The inclusion criteria were breast cancer survivors who had completed their primary treatment and were either scheduled for or currently undergoing follow-up examinations; aged between 18 and 70 years; diagnosed with breast cancer at stages I, II, or III; with a BMI≥25 kg/m² and <40 kg/m²; and capable of using a mobile app, as this was likely a component of the study methodology or follow-up process. All participants were required to sign a research consent form, acknowledge their voluntary participation, and understand the study’s aims and procedures.

Individuals were excluded from the study if they: (1) were diagnosed with stage IV breast cancer or carcinoma in situ; (2) did not use smartphones; (3) had previous treatment for any cancer, including breast cancer; (4) had multiple organ tumors and recurrent or metastatic cancers; (5) had 2 or more uncontrolled chronic conditions, such as stroke, uncontrolled diabetes mellitus, uncontrolled hypertension, uncontrolled hypercholesterolemia, or uncontrolled psychological disorders; (6) declined to participate in the study; and (7) had communication difficulties impeding participation in the study.

We categorized participants into 3 groups based on the duration of their activities. These groups were defined as hyperactive, active, or inactive. Participants in the hyperactive group engaged in activities for more than 16 weeks. Those in the active group participated in activities for 8‐16 weeks. Finally, the inactive group included participants with less than 8 weeks of activity.

### Procedures

For this study, we developed and implemented a 24-week program, including 5 categorized self-developed educational materials about diet-, exercise-, disease-, lifestyle-, and psychology-related content in the Noom app. The participants were instructed to use the Noom app, a mobile app–based human coaching program. Once familiar with the operation of Noom, the participants were required to log their daily dietary intake and lifestyle habits into the app for 24 weeks. An assigned Noom coach analyzed the data and provided individualized coaching through in-app messages at least once a week, offering feedback on lifestyle habits and dietary modifications [10].

To assess the effectiveness of the mobile app–based human coaching program in managing nutrition and lifestyle habits, the participants underwent physical measurements, clinical laboratory tests, and quality of life (QOL) surveys at the onset of the study and at the 6- and 12-month follow-ups. Anthropometric measurements were assessed using BMI and InBody analysis. For the QOL assessment, we used the EORTC Quality of Life Questionnaire-Core 30 (EORTC QLQ-C30) and QLQ-BR23. These tools were scored according to the European Organisation for Research and Treatment of Cancer (EORTC) scoring manual [11].

### Outcomes

The primary outcome was BMI reduction in the hyperactive group with a 24-week mobile app–based human coaching program through personalized nutrition and lifestyle modifications. Secondary outcomes were weight reduction and improvement in body composition, which were assessed using blood tests, bioimpedance, and QOL.

### Sample Size Calculation and Statistical Analysis

This single-arm study was conducted over 24 weeks. It aimed for a reduction in BMI of at least mean 0.8 (SD 1.45) kg/m^2^ in the hyperactive group, with an α of .05 and 90% power [12]. Considering a dropout rate of 50% after registration to the group of interest and 60% from the group of interest to the hyperactive group, 130 participants were required for this study.

The baseline characteristics were compared among the hyperactive, inactive, and active groups. Comparisons between the 2 groups were performed using the Chi-square test or Fisher exact test and the 2-tailed sample *t* test, depending on the type of variable. Changes in body weight, BMI, and body composition from baseline to 6 months in the hyperactive group were compared using 2-tailed paired *t* tests. One-way ANOVA was performed to determine changes over time for patients with physical measurements and QOL completed in 12 months, and changes at each time point were compared using paired *t* tests. Categorical variables were summarized as frequencies and percentages, continuous variables as means and SDs, and mean differences as difference values and 95% CI. Statistical significance was set at *P*<.05, and the results were analyzed using R version 4.1.2 (R Foundation for Statistical Computing).

### Ethical Considerations

This study was approved by the institutional review board of the National Cancer Center of Korea (NCC2019-0098). All participants provided written informed consent prior to enrollment. The original informed consent obtained for primary data collection included provisions for secondary analysis without requiring additional consent. All experimental protocols were reviewed and approved by the same institutional review board, and all methods were conducted in accordance with relevant ethical guidelines and regulations [13,14]. Study data were anonymized to protect participants’ privacy and confidentiality. During the study period, participants received a small token of appreciation, including partial reimbursement for test-related expenses and access to the paid mobile app.

## Results

**Figure 1.**
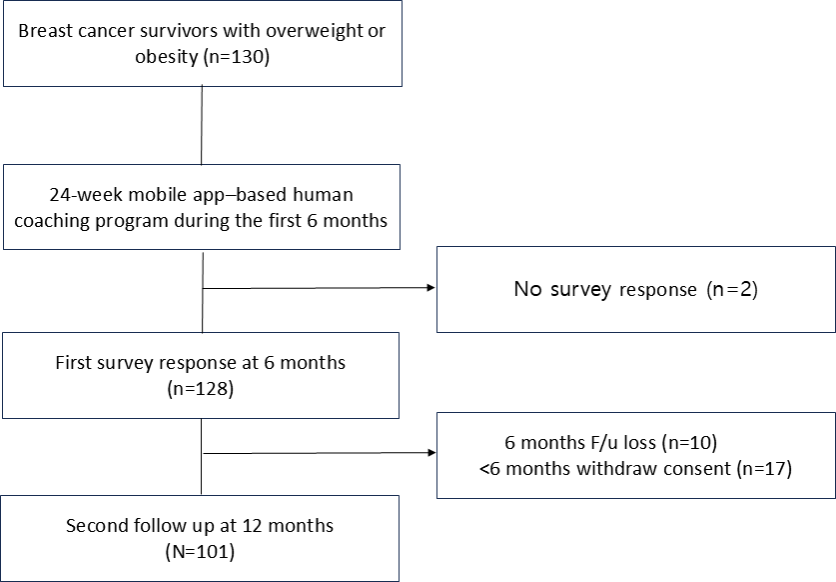
Trials diagram of recruitment and completion.

Between May 2019 and December 2021, 130 participants who met the inclusion criteria were enrolled. A total of 77.5% (101 of the participants completed the study at 6 months follow-up using the 24-week program, and 71.5% (n=93) completed all study 12-month follow-up visits (Figure 1).

The mean age of all participants was 54.90 (SD 7.42) years (54.24, SD 7.84 years for the hyperactive group and 56.27, SD 6.38 years for the inactive and active group). In Baseline, the median BMI for the hyperactive group was 27.14 (IQR 25.20‐35.36), while for the active and inactive groups, it was 27.50 (IQR 25.20‐35.50). Among the participants, 85 (84.2%/101) individuals had a BMI in the range of 25‐29.9, while 16 (15.8%/101) individuals had a BMI of 30 or higher. At baseline, no differences were observed between the groups in terms of BMI, occupational status, clinicopathologic characteristics, and underlying diseases, including hypertension, diabetes mellitus, and hyperlipidemia. Among the participants, 67% (68/101) were in the hyperactive group, and 33% (33/101) were in the inactive and active groups (Table 1).

**Table 1.** Baseline characteristic of study population.

Variables		Total (N=101)	Hyperactive (n=68)	Inactive and active (n=33)	*P* value
Age (year), mean (SD)		54.90 (7.42)	54.24 (7.84)	56.27 (6.38)	.20^a^
Age group (year), n (%)					.47^b^
≤40		5 (5.0)	5 (7)	0 (0)	
41-50		26 (25.7)	18 (26)	8 (24)	
51-60		43 (42.6)	27 (40)	16 (49)	
61-70		27 (26.7)	18 (27)	9 (27)	
BMI, n (%)					.87^c^
25-29.9		85 (84.2)	58 (85)	27 (82)	
≥30		16 (15.8)	10 (15)	6 (18)	
Education, n (%)					.16^b^
Less than a high school diploma		14 (13.9)	10 (15)	4 (12)	
High School Diploma		48 (47.5)	28 (41)	20 (61)	
College degree or higher		39 (38.6)	30 (44)	9 (27)	
Occupation, n (%)					.93^c^
Unemployment		56 (55.5)	37 (54)	19 (58)	
Employment		45 (44.5)	31 (46)	14 (42)	
T^d^ stage, n (%)					.24^b^
1		67 (66.3)	46 (68)	21 (64)	
2		30 (29.7)	21 (31)	9 (27)	
3		4 (4.0)	1 (1)	3 (9)	
N^e^ stage, n (%)					.06^b^
0		68 (67.3)	51 (7)	17 (52)	
1		29 (28.7)	15 (22)	14 (42)	
2		2 (2.0)	1 (1)	1 (3)	
3		2 (2.0)	1 (1)	1 (3)	
Stage, n (%)					.43^b^
I		53 (52.5)	37 (54)	16 (49)	
II		43 (42.5)	29 (43)	14 (42)	
III		5 (5.0)	2 (3)	3 (9)	
ER^f^, n (%)					.77^b^
Negative		15 (14.8)	11 (16)	4 (12)	
Positive		86 (85.2)	57 (84)	29 (88)	
PR^g^, n (%)					.74^c^
Negative		24 (23.8)	15 (22)	9 (27)	
Positive		77 (76.2)	53 (78)	24 (73)	
HER2^h^, n (%)					.77^b^
Negative		86 (85.1)	57 (84)	29 (88)	
Positive		15 (14.9)	11 (16)	4 (12)	
Surgery, n (%)					>.99^b^
BCS^i^		87 (86.1)	58 (85)	29 (88)	
Mastectomy		14 (13.9)	10 (15)	4 (12)	
Chemotherapy, n (%)					.53^c^
Done		49 (48.5)	31 (46)	18 (55)	
Not done		52 (51.5)	37 (54)	15 (45)	
Radiotherapy, n (%)					.71^b^
Done		92 (91.1)	61 (90)	31 (94)	
Not done		9 (8.9)	7 (10)	2 (6)	
Antihormonal therapy, n (%)					.54^b^
Done		87 (86.1)	57 (84)	30 (91)	
Not done		14 (13.9)	11 (16)	3 (9)	
HTN^j^, n (%)					.11^c^
Yes		28 (27.7)	15 (22)	13 (39)	
No		73 (72.3)	53 (78)	20 (61)	
DM^k^, n (%)					.54^b^
Yes		13 (12.9)	10 (15)	3 (9)	
No		88 (87.1)	58 (85)	30 (91)	
Hyperlipidemia, n (%)					.70^c^
Yes		19 (18.8)	14 (21)	5 (15)	
No		82 (81.2)	54 (79)	28 (85)	

a *t* test.

b Fisher exact test, *P*<.05: significance levels.

c *χ*2 test.

d T: tumor.

e N: node.

f ER: estrogen receptor.

g PR: progesterone receptor.

h HER2: human epidermal growth factor receptor 2.

i BCS: breast-conserving surgery.

j HTN: hypertension.

k DM: diabetes mellitus.

At 6 months, 68 patients in the hyperactive group achieved an average weight loss of 1.96 kg and a mean decrease in BMI of 0.86 (SD 1.22) kg/m^2^. In addition, on average, skeletal muscle mass increased by 0.75 (SD 2.6) kg, and body fat percentage decreased by 3.58% (SD 6.88). Furthermore, the waist-hip ratio decreased by 0.04, and the visceral fat area decreased by 23 cm² (Table 2).

When comparing the hyperactive group with the active and inactive groups over 6 months, there was a statistically significant decrease in weight, BMI, total cholesterol level, LDL-cholesterol level, body fat mass (BFM, kg), percent body fat (PBF, %), and visceral fat area (VFA; Table S1 in Multimedia Appendix 1).

**Figure 2.**
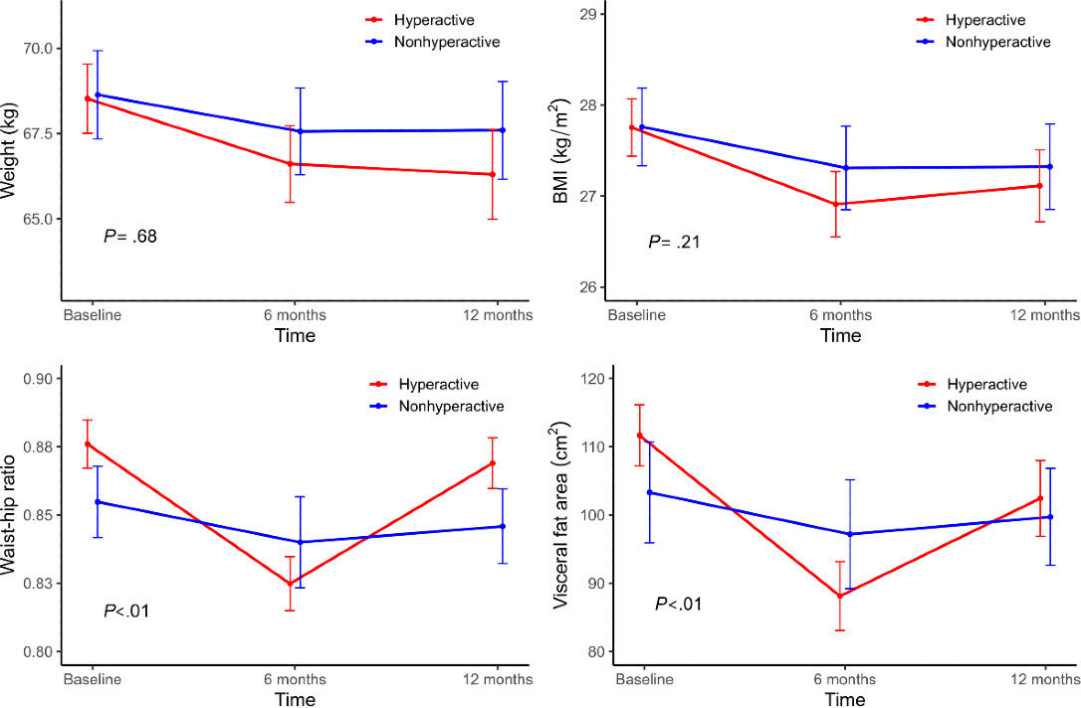
Weight, BMI, and body composition change of hyperactive versus nonhyperactive (inactive and active) group at 6 months and 12 months.

At 12 months, 61 participants in the hyperactivity group had completed the program. Compared with baseline, there were statistically significant decreases in weight, BMI, fasting blood sugar (FBS) level, triglyceride level, skeletal muscle mass, BFM, PBF, waist-to-hip ratio, and VFA at 6 months. Furthermore, at 12 months, there were significant decreases in weight, BMI, FBS, triglyceride, BFM, PBF, and VFA. However, when examining the differences in changes between 6 and 12 months for this hyperactive group, the weight change decreased by 0.31 kg and the BMI change increased by 0.2 kg/m^2^ without statistical significance. Furthermore, the differences in waist-to-hip ratio and VFA showed statistically significant increases of 0.04 (*P*<.01) and 14.33 cm² (*P*=.01), respectively (Figure 2, Table 3).

When comparing QOL using the EORTC QLQ-C30 and EORTC QLQ-BR23 questionnaires among those in the hyperactive group who completed the 12-month program, there was a statistically significant improvement in symptoms related to nausea and vomiting among the symptom scales. During the 6-month program, there were significant improvements in nausea and vomiting, constipation, body image, arm symptoms, and breast symptoms. However, at 12 months, only nausea and vomiting had significantly improved (Table 4).

**Table 2.** Change of body weight, BMI, and body composition of hyperactive group during 6 months.

Variables	Hyperactive group (n=68)				
	Baseline, mean (SD)	6 months, mean (SD)	Mean difference (SD; 95% CI)	*t* test (*df*=67)	*P* value^a^
Weight (kg)	68.71 (7.83)	66.75 (8.60)	–1.96 (2.87; –2.65 to –1.26)	–5.61	<.01
BMI (kg/m^2^)	27.77 (2.38)	26.91 (2.69)	–0.86 (1.22; –1.15 to –0.56)	–5.8	<.01
SBP^b^ (mmHg)	132.65 (13.07)	129.66 (12.85)	–2.99 (14.31; –6.45 to 0.48)	–1.72	.09
DBP^c^ (mmHg)	78.43 (9.99)	76.57 (8.41)	–1.85 (9.76; –4.22 to 0.51)	–1.57	.12
FBS^d^ (mg/dL)	114.44 (29.53)	105.97 (16.67)	–8.47 (26.09; –14.79 to –2.16)	–2.68	.09
HbA_1c_^e^ (%)	6.07 (0.68)	6.01 (0.54)	–0.06 (0.44; –0.15 to 0.04)	–10.97	.23
Total cholesterol (mg/dL)	192.78 (42.33)	191.9 (38.42)	–0.88 (28.8; –7.85 to 6.09)	–0.25	.80
Triglycerides (mg/dL)	172.68 (93.92)	138.54 (99.29)	–34.13 (98.98; –58.09 to –10.17)	–2.84	.01
HDL^f^ -cholesterol (mg/dL)	56.18 (13.90)	57.76 (13.62)	1.59 (9.83; –0.79 to 3.97)	1.33	.19
LDL^g^ -cholesterol (mg/dL)	110.49 (34.86)	109.13 (32.45)	–1.35 (25.84; –7.61 to 4.90)	–0.43	.67
Skeletal muscle mass (kg)	23.85 (3.03)	24.6 (4.00)	0.75 (2.6; 0.12 to 1.38)	2.38	.02
Body fat mass (kg)	25.18 (5.23)	22.17 (6.90)	–3.01 (4.94; –4.20 to –1.81)	–5.02	<.01
Percent body fat (%)	36.47 (4.64)	32.89 (7.97)	–3.58 (6.88; –5.25 to –1.92)	–4.3	<.01
Waist-hip ratio	0.88 (0.07)	0.84 (0.14)	–0.04 (0.15; –0.07 to –0.002)	–2.13	.04
VFA^h^ (cm^2^)	113.1 (34.57)	90.09 (41.3)	–23.00 (40.71; –32.86 to –13.15)	–4.66	<.01

a 2-tailed paired *t* test, *P*<.05: significance levels.

b SBP: systolic blood pressure.

c DBP: diastolic blood pressure.

d FBS: Fasting blood sugar.

e HbA_1c_: glycosylated hemoglobin A_1c_.

f HDL: high-density lipoprotein.

g LDL: low-density lipoprotein.

h VFA: visceral fat area.

**Table 3.** Change of body weight, BMI, and body composition of the hyperactive group at 6 and 12 months.

Variables	Hyperactive (n=61)			F (*df*=2, 180)	*P*^a^ value	6 months-Baseline			12 months-Baseline			12 months-6 months		
	Baseline	6 months	12 months			Mean difference (SD; 95% CI)	*t* test (*df*=60)	*P*^b^ value	Mean difference (SD; 95% CI)	*t* test (*df*=60)	*P*^b^ value	Mean difference (SD; 95% CI)	*t* test (*df*=60)	*P*^b^ value
Weight (kg)	68.52 (7.88)	66.61 (8.78)	66.3 (10.25)	1.52	.22	–1.91 (2.91; –2.65 to –1.17)	–5.13	<.01	–2.22 (5.68; –3.67 to –0.77)	–3.05	<.01	–0.31 (5.8; –1.79 to 1.17)	–0.42	.68
BMI (kg/m^2^)	27.75 (2.45)	26.91 (2.8)	27.11 (3.08)	1.08	.34	–0.84 (1.24; –1.16 to –0.53)	–5.31	<.01	–0.64 (1.59; –1.05 to –0.23)	–3.14	<.01	0.2 (1.27; –0.12 to 0.53)	1.25	.22
SBP^c^ (mmHg)	132.18 (13.43)	129.44 (13.22)	131.98 (11.87)	0.86	.42	–2.74 (14.76; –6.52 to 1.04)	–1.45	.15	–0.2 (14.87; −4.01 to 3.61)	–0.1	.92	2.54 (12.81; –0.74 to 5.82)	1.55	.13
DBP^d^ (mmHg)	78.64 (9.92)	76.69 (8.62)	76.75 (8.63)	0.91	.40	–1.95 (10.1; –4.54 to 0.64)	–1.51	.14	–1.89 (10.71; –4.63 to 0.86)	–1.37	.17	0.07 (8.71; –2.16 to 2.3)	0.06	.95
FBS^e^ (mg/dL)	114.08 (30.44)	106.11 (16.69)	105.59 (21.21)	2.5	.08	–7.97 (26.75; –14.82 to –1.12)	–2.33	.02	–8.49 (25.33; –14.98 to –2)	–2.62	.01	–0.52 (17.4; –4.98 to 3.93)	–0.24	.81
HbA_1c_^f^ (%)	6.04 (0.69)	5.99 (0.54)	6.10 (0.70)	0.48	.62	–0.05 (0.41; –0.15 to 0.05)	–0.98	.33	0.06 (0.63; –0.1 to 0.22)	0.8	.43	0.11 (0.49; –0.01 to 0.24)	1.83	.07
Total cholesterol (mg/dL)	190.98 (41.99)	190.51 (38.55)	186.3 (39.55)	0.25	.78	–0.48 (28.94; –7.89 to 6.94)	–0.13	.90	–4.69 (35.15; –13.69 to 4.31)	–1.04	.30	–4.21 (23.6; –10.26 to 1.83)	–1.39	.17
Triglycerides (mg/dL)	174.02 (92.65)	132.02 (69.74)	135.72 (65.26)	5.59	<.01	–42 ( 84.62; –63.67 to –20.33)	–3.88	<.01	–38.3 (79.04; –58.54 to –18.05)	–3.78	<.01	3.7 (60.89; –11.89 to 19.3)	0.48	.64
HDL^g^ -cholesterol (mg/dL)	55.77 (13.49)	57.79 (13.03)	57.57 (14.04)	0.41	.66	2.02 (9.99; –0.54 to 4.58)	1.58	.12	1.8 (10.97; –1.01 to 4.61)	1.28	.20	–0.21 (8.98; –2.51 to 2.09)	–0.19	.85
LDL^h^ -cholesterol (mg/dL)	109.64 (35.16)	109.02 (32.92)	107.8 (34.99)	0.05	.96	–0.62 (26.54; –7.42 to 6.17)	–0.18	.86	–1.84 (30.98; –9.77 to 6.1)	–0.46	.65	–1.21 (21.23; –6.65 to 4.22)	–0.45	.66
Skeletal muscle mass (kg)	23.81 (3.08)	24.73 (3.99)	24.23 (3.21)	1.09	.34	0.92 (2.52; 0.28 to 1.56)	2.85	.01	0.42 (1.97; –0.09 to 0.92)	1.65	.10	–0.5 (2.85; –1.23 to 0.23)	–1.37	.17
Body fat mass (kg)	25.06 (5.28)	21.79 (6.75)	23.04 (7.2)	3.98	.02	–3.27 (4.8; –4.5 to –2.04)	–5.33	<.01	–2.02 (4.5; –3.17 to –0.87)	–3.51	<.01	1.25 (5.65; –0.19 to 2.7)	1.73	.09
Percent body fat (%)	36.39 (4.72)	32.36 (7.53)	33.78 (6.85)	6.07	<.01	–4.03 (6.53; –5.7 to –2.36)	–4.82	<.01	–2.61 (5.75; –4.08 to –1.13)	–3.54	<.01	1.42 (7.49; –0.5 to 3.34)	1.48	.14
Waist-hip ratio	0.88 (0.07)	0.82 (0.08)	0.87 (0.07)	8.58	<.01	–0.05 (0.09; –0.08 to –0.03)	–4.27	<.01	–0.01 (0.06; –0.02 to 0.01)	–0.85	.40	0.04 (0.1; 0.02 to 0.07)	3.24	<.01
VFA^i^ (cm^2^)	111.68 (35.05)	88.12 (39.47)	102.45 (43.44)	5.52	<.01	–23.56 (39.28; –33.62 to –13.5)	–4.68	<.01	–9.23 (29.27; –16.72 to –1.73)	–2.46	.02	14.33 (42.95; 3.33 to 25.33)	2.61	.01

a *P*: one way ANOVA.

b *P*: 2-tailed paired T-test, *P*<.05: significance levels.

c SBP: systolic blood pressure.

d DBP: diastolic blood pressure.

e FBS: fasting blood sugar.

f HbA_1c_: glycosylated hemoglobin A_1c_.

g HDL: high-density lipoprotein.

h LDL: low-density lipoprotein.

i VFA: visceral fat area.

**Table 4.** Comparison of quality of life in hyperactive group (n=61) through 12 months.

Variables	Hyperactive (n=61)			F (*df*=2, 180)	*P*^a^ value	Hyperactive								
	Baseline	6 months	12 months			6 months-Baseline			12 months-Baseline			12 months-6 months		
	mean (SD)	mean (SD)	mean (SD)			Mean difference (SD; 95% CI)	*t* test (*df*=60)	*P*^b^ value	Mean difference (SD; 95% CI)	*t* test (*df*=60)	*P*^b^ value	Mean difference (SD; 95% CI)	*t* test (*df*=60)	*P*^b^ value
EORTC QLQ-C30														
Global health status	66.94 (19.06)	67.49 (18.43)	70.9 (18.92)	0.8	.45	0.55 (19.12; –4.35 to 5.44)	0.22	.82	3.96 (21.5; –1.54 to 9.47)	1.44	.16	3.42 (21.96; –2.21 to 9.04)	1.21	.23
Functional scales														
Physical functioning	82.62 (10.91)	84.04 (9.98)	83.39 (13.31)	0.23	.79	1.42 (10.26; –1.21 to 4.05)	1.08	.28	0.77 (12.48; –2.43 to 3.96)	0.48	.63	–0.66 (12.45; –3.85 to 2.53)	–0.41	.68
Role functioning	84.43 (19.92)	88.8 (17.41)	89.34 (15.82)	1.4	.25	4.37 (17.72; –0.17 to 8.91)	1.93	.06	4.92 (18.6; 0.16 to 9.68)	2.07	.04	0.55 (19.48; –4.44 to 5.53)	0.22	.83
Emotional functioning	82.1 (17.54)	78.96 (21.87)	80.87 (16.41)	0.43	.65	–3.14 (20.48; –8.39 to 2.1)	–1.2	.24	–1.23 (14.01; –4.82 to 2.36)	–0.69	.50	1.91 ( 20.21; –3.26 to 7.09)	0.74	.46
Cognitive functioning	79.51 (17.58)	80.6 (15.57)	79.51 (18.61)	0.08	.92	1.09 (16.35; –3.09 to 5.28)	0.52	.60	0 (12.91; –3.31 to 3.31)	0	>.99	1.09 (16.35; –5.28 to 3.09)	–0.52	.60
Social functioning	89.07 (15.48)	92.35 (15.39)	90.44 (16.52)	0.66	.52	3.28 (16.05; –0.83 to 7.39)	1.6	.12	1.37 (18.58; –3.39 to 6.13)	0.57	.57	–1.91 (20.43; –7.15 to 3.32)	–0.73	.47
Symptom scales														
Fatigue	30.42 (20.17)	28.42 (17.75)	27.69 (16.31)	0.37	.69	–2 (17.63; –6.52 to 2.51)	–0.89	.38	–2.73 (14.72; –6.5 to 1.04)	–1.45	.15	–0.73 (15.43; –4.68 to 3.22)	–0.37	.71
Nausea / Vomiting	7.38 (13.45)	2.73 (6.93)	3.01 (9.38)	3.92	.02	–4.64 (13.99; –8.23 to –1.06)	–2.59	.01	–4.37 (14.24; –8.02 to –0.72)	–2.4	.02	0.27 (8.33; –1.86 to 2.41)	0.26	.80
Pain	19.67 (24.25)	19.95 (17.7)	18.03 (22.63)	0.14	.87	0.27 (18.88; –4.56 to 5.11)	0.11	.91	–1.64 (21.45; –7.13 to 3.86)	–0.6	.55	–1.91 (22.38–7.64 to 3.82)	–0.67	.51
Dyspnea	16.39 (23.27)	14.21 (19.68)	16.39 (22.46)	0.2	.82	–2.19 (27.13; –9.13 to 4.76)	–0.63	.53	0 (26.53; –6.79 to 6.79)	0	>.99	2.19 (21.83; –3.41 to 7.78)	0.78	.44
Insomnia	33.88 (30.73)	39.34 (33.61)	37.16 (32.26)	0.44	.64	5.46 (32.87; –2.96 to 13.88)	1.3	.20	3.28 ( 27.02; –3.64 to 10.2)	0.95	.35	–2.19 (30.35; –9.96 to 5.59)	–0.56	.58
Appetite loss	7.65 (21.42)	8.2 (17.9)	6.01 (15.53)	0.23	.79	0.55 (26.17; –6.16 to 7.25)	0.16	.87	–1.64 (25.41; –8.15 to 4.87)	–0.5	.62	–2.19 (20.97; –7.56 to 3.18)	–0.81	.42
Constipation	23.5 (28.77)	14.21 (20.6)	19.67 (25.37)	0.72	.49	–9.29 (22.88; –15.15 to –3.43)	–3.17	<.01	–3.83 (25.16; –10.27 to 2.62)	–1.19	.24	5.46 ( 24.48; –0.81 to 11.73)	1.74	.09
Diarrhea	9.29 (16.25)	9.84 (17.58)	6.56 (14.68)	2.1	.12	0.55 (17.73; –4 to 5.09)	0.24	.81	–2.73 (15.27; –6.64 to 1.18)	–1.4	.17	–3.28 (18.96; –8.13 to 1.58)	–1.35	.18
Financial problems	7.1 (17.34)	6.01 (16.68)	7.65 (16.55)	0.15	.86	–1.09 (20.15; –6.25 to 4.07)	–0.42	.67	0.55 (16.66–3.72 to 4.81)	0.26	.80	1.64 (18.68; –3.15 to 6.42)	0.69	.50
QLQ-C30 Summary Score	82.27 (10.59)	83.68 (9.14)	83.77 (9.69)	0.45	.64	1.41 (9.15; –0.93 to 3.75)	1.2	.23	1.5 (8.33; –0.63 to 3.63)	1.41	.17	0.09 (9.97; –2.46 to 2.64)	0.07	.94
EORTC QLQ-BR23														
Functional scales														
Body image	67.35 (27.19)	75 (25.69)	71.45 (25.43)	1.31	.27	7.65 (23.49; 1.64 to 13.67)	2.54	.01	4.1 (24.04; –2.06 to 10.25)	1.33	.19	–3.55 (20.61; –8.83 to 1.73)	–1.35	.18
Future Perspective	50.82 (28.94)	58.47 (27.66)	56.28 (28.25)	1.18	.31	7.65 (33.55; –0.94 to 16.24)	1.78	.08	5.46 (32.31; –2.81 to 13.74)	1.32	.19	–2.19 (27.8; –9.31 to 4.93)	–0.61	.54
Symptom scales														
Arm symptoms	29.69 (21.63)	22.22 (19.88)	26.78 (20.92)	1.99	.14	–7.47 (17.77; –12.02 to –2.92)	–3.28	.00	–2.91 (23.03; –8.81 to 2.98)	–0.99	.33	4.55 (23.07; –1.36 to 10.46)	1.54	.13
Breast symptoms	14.48 (15.73)	10.79 (10.69)	12.57 (12.42)	1.21	.30	–3.69 ( 14.23; –7.33 to –0.04)	–2.02	.05	–1.91 (13.38; –5.34 to 1.52)	–1.12	.27	1.78 (10.33; –0.87 to 4.42)	1.34	.18
Systemic therapy	23.26 (13.53)	22.56 (12.83)	23.11 (12.83)	0.05	.95	–0.7 (11.66; –3.69 to 2.28)	–0.47	.64	–0.16 (10.06; –2.73 to 2.42)	–0.12	.90	0.55 (9.92; –1.99 to 3.09)	0.43	.67

a *P*: one-way ANOVA.

b *P*: Paired *t* test, *P*<.05: significance levels.

## Discussion

### Principal Findings

This study showed that a 24-week mobile app–based human coaching program for breast cancer survivors was effective in reducing excessive weight and obesity. The hyperactive group lost 1.96 kg and 0.86 kg/m^2^ at 6 months. Also, the hyperactive group showed improved QOL at 6 months. At the 12-month follow-up, weight and BMI tended to return toward baseline levels, suggesting that the intervention period may need to be extended for sustained effects. This highlights the importance of considering long-term strategies to maintain the benefits observed during the intervention. The significant impact of structured exercise programs is evident in weight management, body composition improvements, and overall physical and mental well-being.

A study found that combined aerobic and resistance training was the most effective exercise modality for improving cardiometabolic health markers. The findings suggest a synergistic effect when integrating multiple exercise types, leading to enhanced body composition and metabolic health outcomes [15]. Another review study found that regular, moderate-to-high-intensity aerobic exercise, especially when combined with weight loss and dietary modifications, can lead to sustained improvements in HDL-C levels. The authors recommend that individuals with overweight and obesity engage in such exercise regimens to enhance their HDL-C levels and overall health [16]. Other exercises, including yoga and Pilates, led to significant improvements in anthropometric measures, body composition, glucose and lipid metabolism, and blood pressure among women who were sedentary, overweight, or had obesity [17-19]. In our study, we observed that participants who actively engaged with the Noom platform experienced significant improvements in body composition, including reductions in body weight and BMI.

Maintaining a healthy weight through diet and exercise can help regulate hormone levels and improve overall health, potentially enhancing survival rates and reducing recurrence. In a previous study, a 16-week monitored program of aerobic and resistance exercises designed to target metabolic syndrome significantly improved QOL, depression symptoms, fatigue levels, and physical fitness in ethnically diverse, inactive, overweight survivors with obesity or breast cancer. These improvements remained evident at the follow-up 3 months later [20].

Our study showed that body weight, FBS, and triglyceride levels decreased over 6 months, which was maintained for up to 12 months; however, no further change was observed between the 6- and 12-month marks. Regarding BMI, an improvement was observed during the initial 6 months of using the Noom app. However, when Noom was discontinued, the following 6‐12 months showed a reversal to baseline levels. The limitations in maintaining a diet over an extended period may be influenced by various factors, including individual dietary habits, social and cultural influences, psychological factors, and environmental barriers that often make long-term diet adherence challenging.

Being excessively overweight, or people with obesity, is linked to a higher risk of developing breast cancer, especially in postmenopausal women [21]. A study showed significantly poorer outcomes, including overall survival (OS) and disease-free survival (DFS), in patients with severe obesity (BMI≥40 kg/m²). This contrast was not observed in patients who have moderate obesity (BMI 35.0‐39.9 kg/m²), those with slight obesity (BMI 30.0‐34.9 kg/m²), or those with excessive weight (BMI 25.0‐29.9 kg/m²) when compared with those who are underweight or of normal weight (BMI<25.0 kg/m²) with early breast cancer [22]. A total of 301 menopausal women with breast cancer were studied. The findings showed an adjusted OR of 1.37 (95% CI 0.73-2.56) for patients with a BMI of 27 kg/m^2^ or more compared to those with a BMI under 27 kg/m^2^. They suggest that patients with obesity had poorer survival rates [23]. A meta-analysis of 43 other studies indicated that women with obesity and breast cancer had lower survival rates than women without obesity. This was consistent for OS (hazard ratio 1.33 95% CI 1.21-1.47) and breast cancer–specific survival (hazard ratio 1.33; 95% CI 1.19-1.50) [2].

Breast cancer survivors frequently experience cancer-related symptoms, including cognitive difficulties, pain, insomnia, and urinary incontinence, with 65% experiencing ongoing pain [24-26]. Weight management improves the QOL of breast cancer survivors by alleviating side effects, improving mobility, and boosting mental health. A comprehensive randomized controlled trial involving a diverse cohort of breast cancer survivors who are excessively overweight or people with obesity found that, while an intensive, group-based weight loss intervention yielded positive results in reducing weight, it led to only marginal improvements in vitality and transient enhancements in physical functioning and symptomatology [27]. Our study yielded similar results. In the hyperactive group, various physical symptoms, including body image, improved during the first 6 months. However, only the alleviation of nausea and vomiting was sustained for more than 12 months.

Mobile interventions for weight management programs are widely used in various diseases, such as diabetes, heart disease, and obesity, especially those where weight management is crucial [9,10,12]. Among patients with type 2 diabetes, those who completed the program experienced a significant weight reduction of 5.6% (with a SE of 0.81; *P*<.001) after 6 months. They maintained a weight loss of 4.7% (with an SE of 0.88; *P*<.001) at the 12-month mark. In contrast, the control group showed a negligible weight loss of 0.15% at 6 months (SE 0.64; *P*=.85) and a slight weight gain of 0.33% (SE 0.70; *P*=.63) at 12 months [28]. In the systematic review, 7 papers [29, 30, 31, 32, 33, 34, 35] met the inclusion criteria. The most commonly observed measure in these studies was the change in participants’ weight, which was noted in 57% of the studies. In addition, most studies (71%) found statistically significant improvements in at least one area, including weight loss, increased physical activity, dietary habits, reduction in BMI, reduced waist circumference, decreased intake of sugar-sweetened beverages, reduced screen time, and measures of satisfaction or acceptability [36]. In our study as well, we observed improvements in weight management, physical activity, and QOL in the hyperactive group when the device was applied. The use of these devices is clinically significant for patients with breast cancer. Many of these patients consistently focus on dietary habits and weight management. Therefore, smartphone-based interventions that offer enhanced accessibility could improve patient care and reduce the risk of obesity. Another study showed the impact of the Noom mobile behavior change program on weight loss among breast cancer survivors, reporting a mean weight loss of 4.8 (SD 4.4) kg (5.6% of initial body weight) over 26 weeks, with increased physical activity levels and improved body image. Their study primarily focused on self-reported weight changes and app engagement [37]. In contrast, our study extends beyond weight loss by assessing changes in body composition, including metabolic health markers, rather than focusing solely on BMI or weight. In addition, a key strength of our study is the postintervention follow-up, which examines whether the effects of app usage are sustained after the cessation of the program. This provides valuable insights into the long-term effectiveness of mobile health interventions in breast cancer survivors.

### Limitation

However, this study has a few limitations. First, this study regards the lack of information to identify the association of this program with OS or DFS in the participants. Further follow-up research is necessary to investigate the mobile health intervention value for OS and DFS. Second, this study has a relatively small number of participants with obesity, defined as BMI >30. Owing to the potential for different patterns of change depending on the degree of obesity, future studies may need to be stratified by similar proportions to address this variable. This study was a preliminary, single-arm prospective study designed to evaluate the efficacy of an application program in modifying body composition. However, as the analysis was conducted only on patients with hyperactivity, there is a potential for selection bias. Further research, including a randomized controlled trial, is needed to validate these findings.

### Implications

This study demonstrates a shift from the traditional, resource-intensive approaches to nutritional interventions and lifestyle modifications. By using the mobile app–based Noom platform, this study provided a structured and accessible method for dietary tracking and lifestyle management. Furthermore, it implemented personalized nutrition and lifestyle adjustments through human coaching. This study was able to realize improvements in nutrition and lifestyle habits, ultimately contributing to improvement in QOL among breast cancer survivors.

### Conclusions

Combating obesity and encouraging healthy lifestyle habits are essential components of preventive and therapeutic strategies for breast cancer. Mobile health intervention strategies could prove beneficial in various programs by providing information and support for the self-monitoring of survivors within supervised settings.

## Supplementary material

10.2196/64846 Multimedia Appendix 1 Comparison between the hyperactive group and the combined active and inactive groups over 6 months.

## Abbreviations

BFM: body fat mass
BR 23: Breast Cancer Module (23 items)
DFS: disease-free survival
EORTC: European Organisation for Research and Treatment of Cancer
EORTC QLQ-C30: European Organisation for Research and Treatment of Cancer Quality of Life Questionnaire-Core 30
FBS: fasting blood sugar
OR: odds ratio
OS: overall survival
PBF: percent body fat
QOL: quality of life
VFA: visceral fat area
