# Supplementary material for: Impact of a 24-Week Mobile App–Based Human Coaching Program on Body Composition and Lipid Metabolism in Breast Cancer Survivors With Overweight or Obesity: Single-Arm Prospective Cohort Study
Source: JMIR Mhealth Uhealth. 2025 Sep 24;13:e64846. doi: 10.2196/64846 (PMC12459734; doi:10.2196/64846)
Supplement: Multimedia Appendix 1 [file mhealth-v13-e64846-s001.docx]

**Supplemental Table 1. Comparison between the Hyperactive group and the combined Active & Inactive groups over 6 months** BMI: body mass index, SBP: systolic blood pressure, DBP: diastolic blood pressure, FBS: Fasting blood sugar, HbA1C: Glycosylated Hemoglobin A1c, HDL: High-Density Lipoprotein, LDL: Low-Density Lipoprotein, VFA: visceral fat area

**^e^**: Wilcoxon rank sum Text, *P*<.05: significance levels

| **Variables, median (min-max)** | **Hyperactive (N=68)** | | **Active and Inactive (N=33)** | | ***P*^e^** |
| --- | --- | --- | --- | --- | --- |
|  | **Baseline** | **6 months** | **Baseline** | **6 months** |  |
| **Weight (kg)** | 68.60(56.80-88.20) | 65.10(48.40-87.80) | 68.90(56.60-88.00) | 67.50(55.50-89.60) | **0.0267** |
| **BMI (kg/m^2^)** | 27.14(25.20-35.36) | 26.35(21.40-35.20) | 27.50(25.20-35.50) | 27.10(24.10-36.30) | **0.0175** |
| **SBP(mmHg)** | 132.00(104.00-165.00) | 130.00(98.00-163.00) | 127.00(100.00-168.00) | 137.00(104.00-157.00) | 0.0350 |
| **DBP(mmHg)** | 79.00(56.00-102.00) | 76.50(59.00-95.00) | 78.00(59.00-97.00) | 77.00(56.00-93.00) | 0.1920 |
| **FBS (mg/dL)** | 105.50(85.00-264.00) | 103.00(81.00-162.00) | 101.00(84.00-224.00) | 99.00(85.00-189.00) | 0.8393 |
| **HbA1C (%)** | 5.85(5.10-9.10) | 5.80(5.20-7.50) | 5.80(5.10-8.60) | 5.80(5.20-8.30) | 0.0547 |
| **Total cholesterol (mg/dL)** | 188.50(118.00-302.00) | 182.00(127.00-292.00) | 189.00(150.00-275.00) | 194.00(138.00-322.00) | **0.0383** |
| **Triglycerides (mg/dL)** | 155.50(48.00-438.00) | 112.50(30.00-731.00) | 143.00(9.00-433.00) | 134.00(60.00-277.00) | 0.2632 |
| **HDL-cholesterol (mg/dL)** | 55.00(31.00-87.00) | 57.50(35.00-86.00) | 54.00(34.00-75.00) | 54.00(34.00-75.00) | 1.0000 |
| **LDL-cholesterol (mg/dL)** | 105.50(51.00-205.00) | 102.00(56.00-194.00) | 106.00(67.00-194.00) | 113.00(67.00-235.00) | **0.0179** |
| **Skeletal muscle mass(kg)** | 23.55(18.50-38.30) | 23.65(15.70-40.40) | 24.00(19.60-30.40) | 24.00(20.00-39.00) | 0.1329 |
| **Body fat mass (kg)** | 24.35(17.70-41.00) | 21.30(4.00-41.90) | 24.00(8.10-43.50) | 21.90(2.00-45.40) | **0.0028** |
| **Percent body fat (%)** | 36.20(24.30-49.70) | 32.90(6.60-58.50) | 34.90(15.50-49.40) | 34.50(3.00-50.50) | **0.0124** |
| **Waist-hip ratio** | 0.88(0.72-1.05) | 0.83(0.66-1.79) | 0.86(0.75-1.06) | 0.86(0.64-1.01) | 0.0902 |
| **VFA (cm^2^)** | 111.75(46.2-220.7) | 82.80(5.00-225.20) | 101.20(28.60-232.20) | 94.40(5.00-225.90) | **0.0380** |
